# A Rare Case of Malignant Melanoma of the Glans Penis in a Young Male Treated with an Organ-Preserving Surgical Procedure

**DOI:** 10.7759/cureus.71819

**Published:** 2024-10-18

**Authors:** Senthil Kumar, Anurag Sahu, Saravanan Jambunathan, Shantanu Chandrashekhar, Saravanan Kanakasabapathy

**Affiliations:** 1 Urology, SRM Medical College Hospital and Research Centre, Chennai, IND

**Keywords:** glanspenis, inguinal lymph nodes, malignant melanoma, partial penectomy, urethra

## Abstract

Penile melanoma is a rare and highly invasive cancer that is generally diagnosed in the sixth to seventh decades of life. We report a rare case of primary malignant melanoma of the glans penis in a 38-year-old man who presented with a proliferative lesion over the glans with discoloration for six months. The clinical and metastatic workups were unremarkable. Circumcision and biopsy performed outside showed characteristics of squamous cell carcinoma. We treated the patient with a partial penectomy, and the histopathological examination was suggestive of malignant melanoma. A stump revision with bilateral superficial inguinal lymph node dissection was performed. As per the oncologist's opinion, the patient underwent chemotherapy and immunotherapy after surgery. No recurrence or metastasis occurred during the one-year follow-up.

## Introduction

Penile melanoma (PM), comprising less than 2% of penile cancers and 0.1% of all melanomas [1], predominantly occurs on the glans penis (55%), prepuce (28%), shaft (9%), and meatus (8%) [2], with a mean patient age of 52.9 years [3]. Since the first documented case in 1859, only 220 cases have been reported globally [4]. PM originates from the malignant transformation of melanocytes in the neuroectodermal layer, with PMs classified as cutaneous or mucosal based on their location [1,5]. Staging follows a three-stage or American Joint Committee on Cancer tumor, node, metastasis (AJCC TNM) staging system [3,6,7]. This case report presents a young male with malignant melanoma of the glans penis involving the urethral meatus and reviews the literature on the pathogenesis and treatment of PM.

## Case presentation

A 38-year-old Indian man visited the Urology Department with a chief complaint of lesions on his glans penis for the past six months. The lesion was painless, and there were no associated complaints. No urinary or sexual complaints were reported. The patient is sexually active. The patient had no history of severe illness, comorbidities, allergy, or trauma to the penis. The family history was also insignificant. He worked as a laborer and had a low socio-economic level. The patient underwent circumcision with a biopsy from the lesion at an outside hospital, and the biopsy revealed squamous cell carcinoma. 

**Figure 1:**
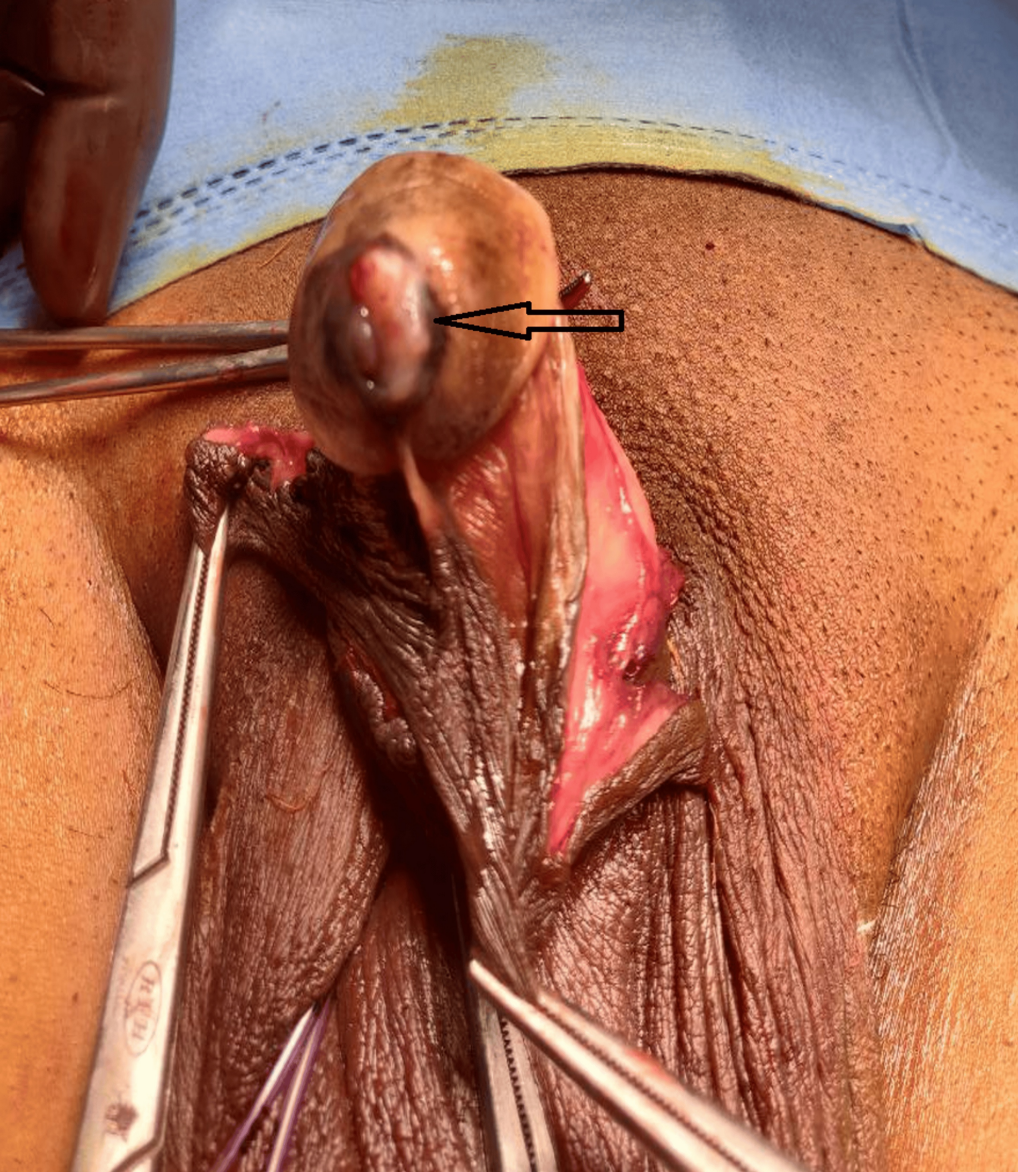
Arrow showing proliferative growth lesion of the glans penis involving urethral meatus with dark brown discoloration

The patient's clinical examination was unremarkable, and local examination revealed proliferative lesions with irregular margins present over the left lateral aspect of the glans penis around the urethral meatus, with dark brown discoloration. The lesion was non-tender and firm in consistency, and no active discharge was observed (Figure 1). No palpable inguinal lymphadenopathy was noted. Testis, scrotum, and prostate examinations were unremarkable.

**Figure 2:**
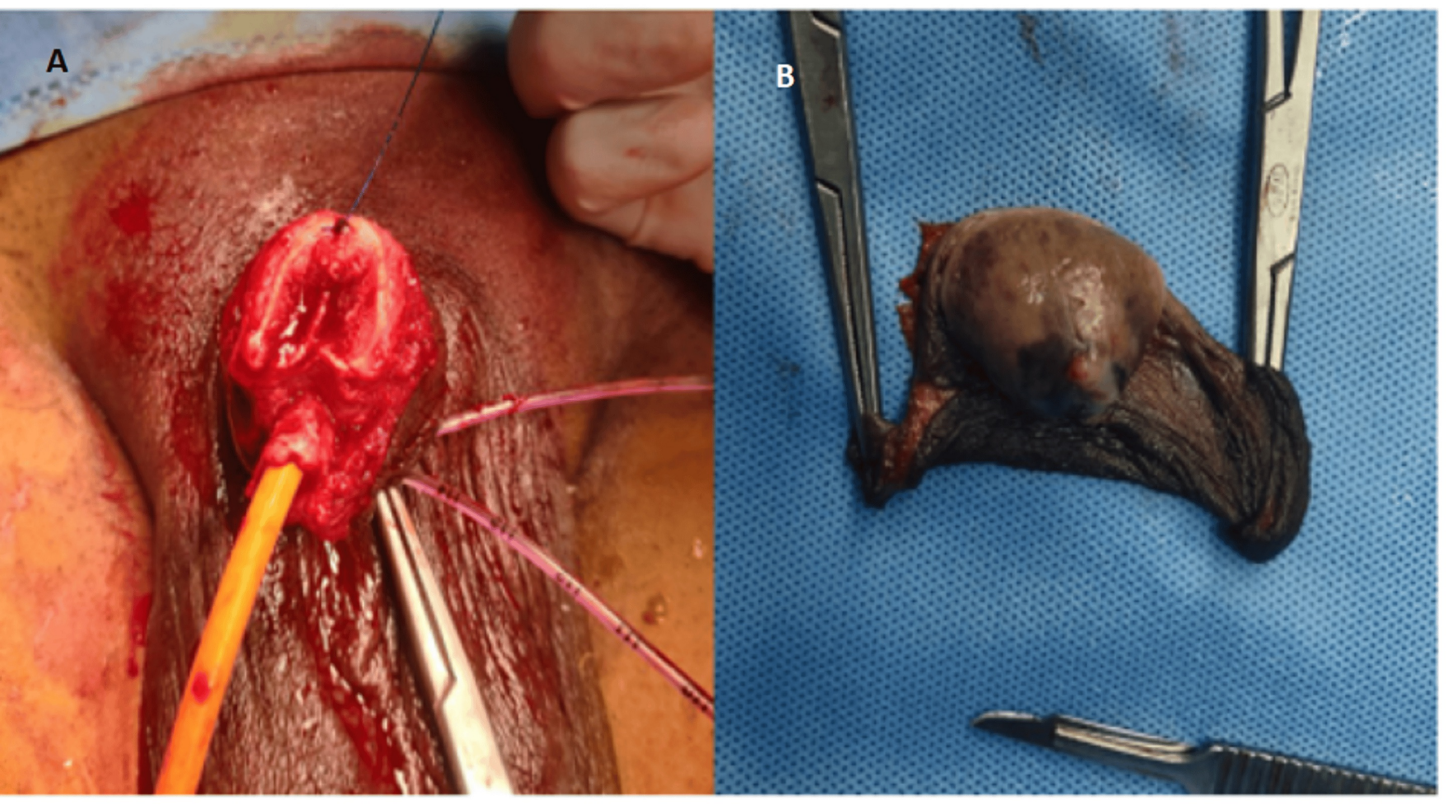
A) Partial penectomy stump. B) Partial penectomy specimen

All routine blood and urine investigations were within normal limits. The contrast-enhanced computed tomography scan of the head, chest, and abdomen did not show evidence of metastatic disease. Subsequently, the patient underwent partial penectomy (Figure 2) based on an outside biopsy report.

**Figure 3:**
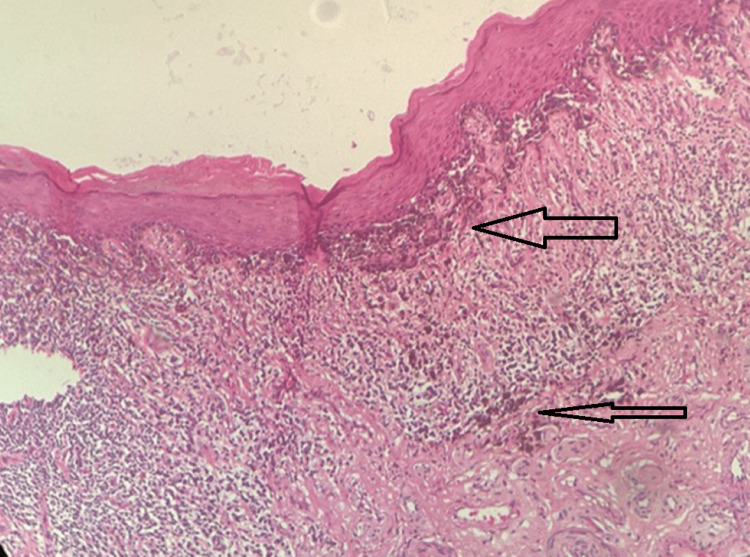
Dense lymphoplasmacytic infiltrates in between the tumor cells in the superficial dermis. No lymphovascular invasion/perineural invasion (H & E 100x)

**Figure 4:**
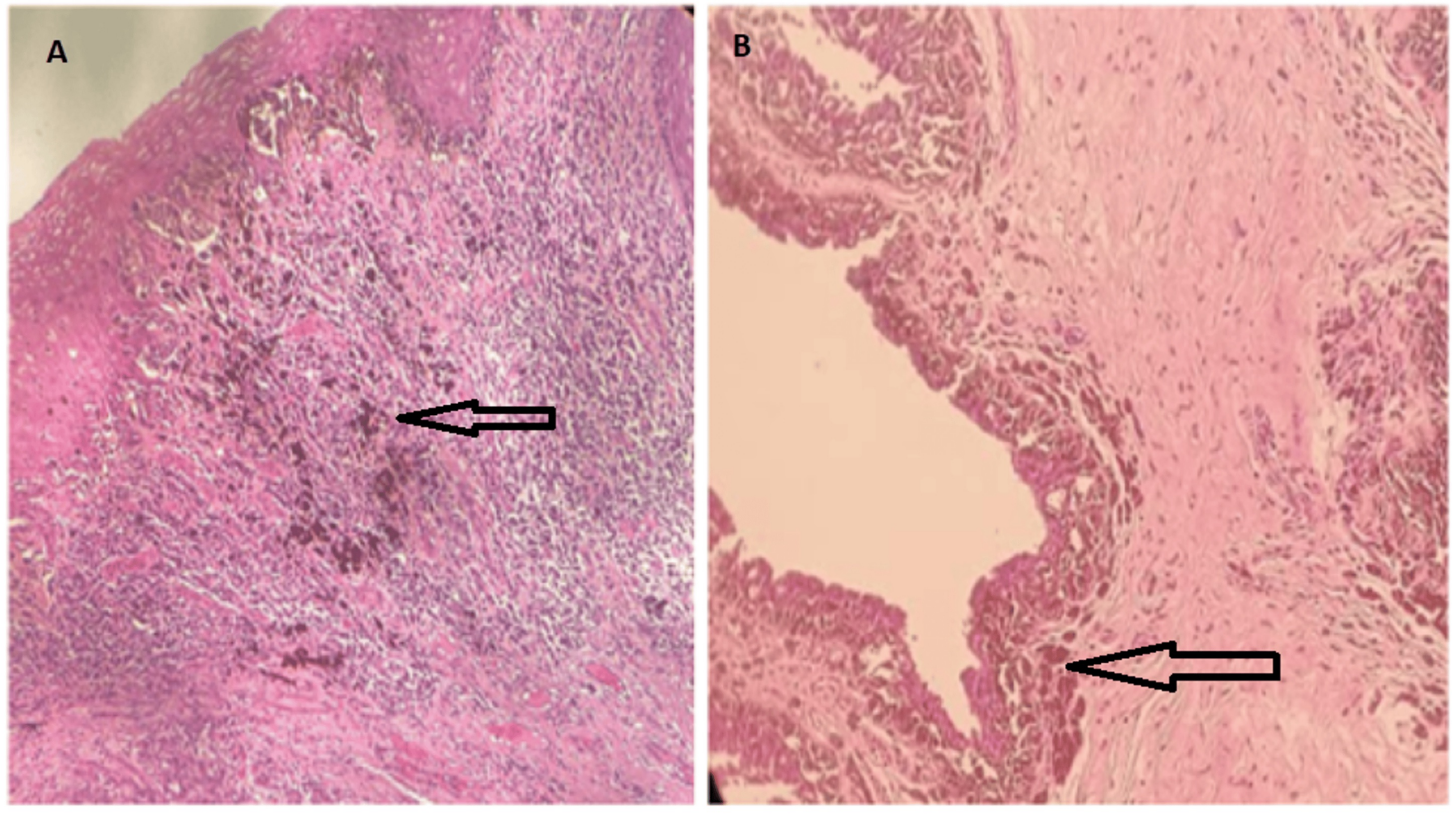
(A) A melanocytic tumor arising from the basal melanocytes of the stratified squamous epithelium, 100x H & E staining. (B) Urothelial lining of urethra is invaded by tumor, 200x H & E staining

Pathological sections of the partial penectomy specimen showed malignant melanoma involving the urethral lining and involvement of the proximal urethral margin (Figures 3-4).

**Figure 5:**
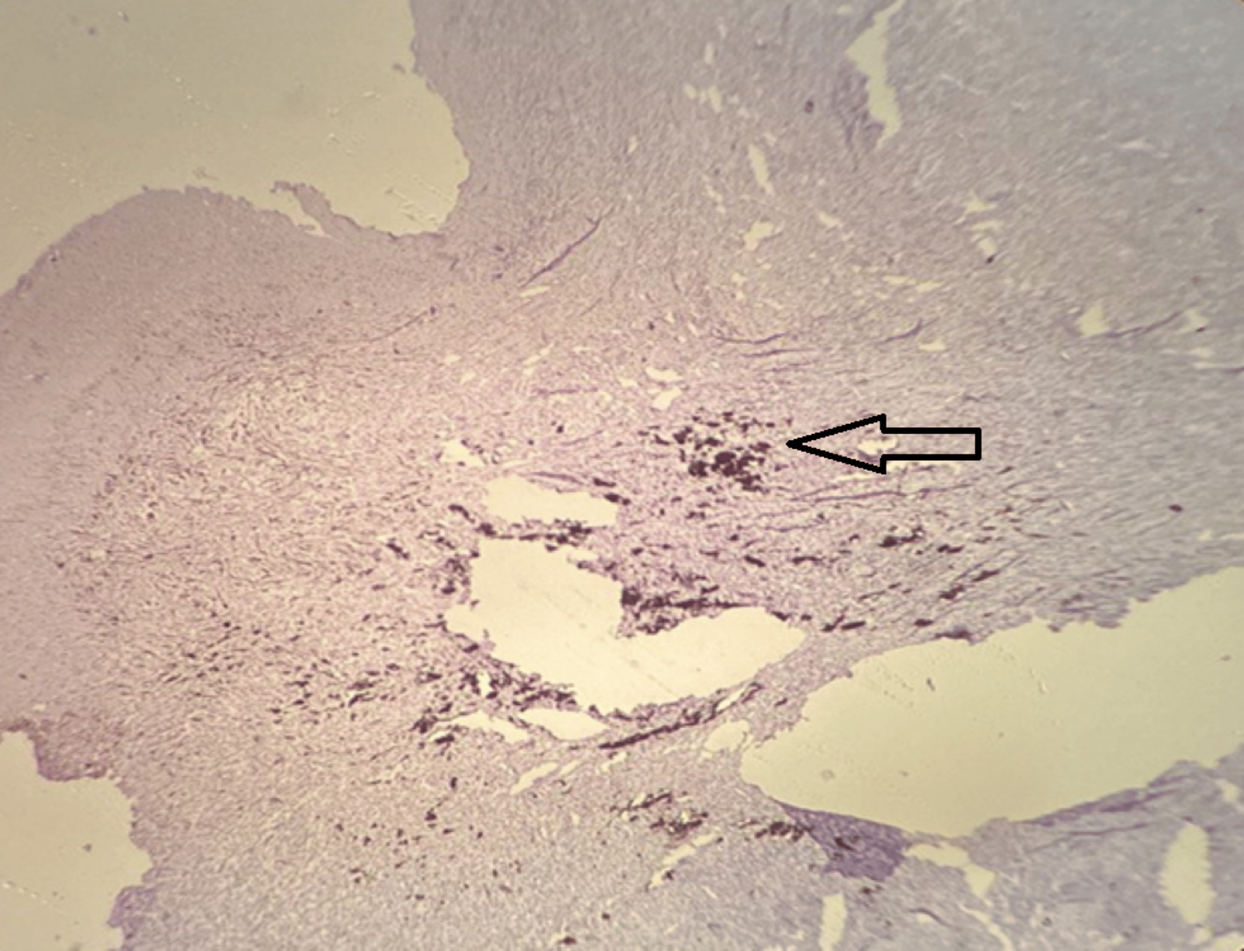
Focal HMB-45 - positive in tumor cells, 200x HMB-45: human melanoma black

**Figure 6:**
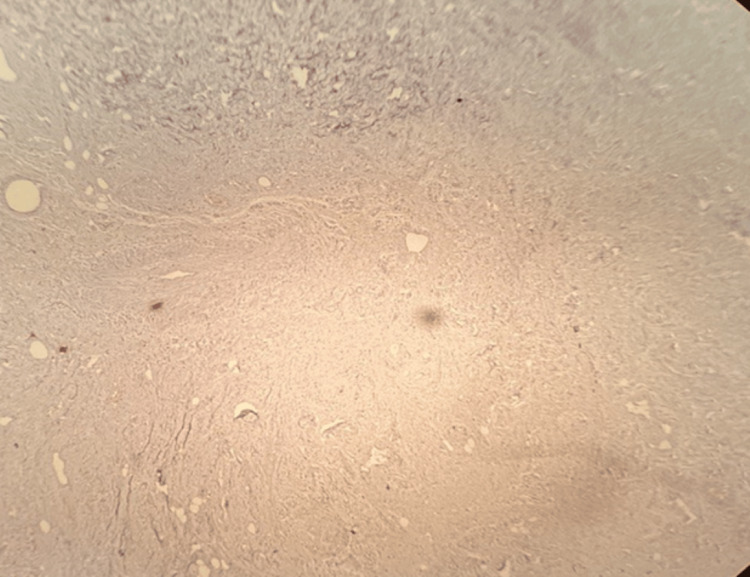
Pan CK - negative, 200x magnification Pan CK: pancytokeratin

**Figure 7:**
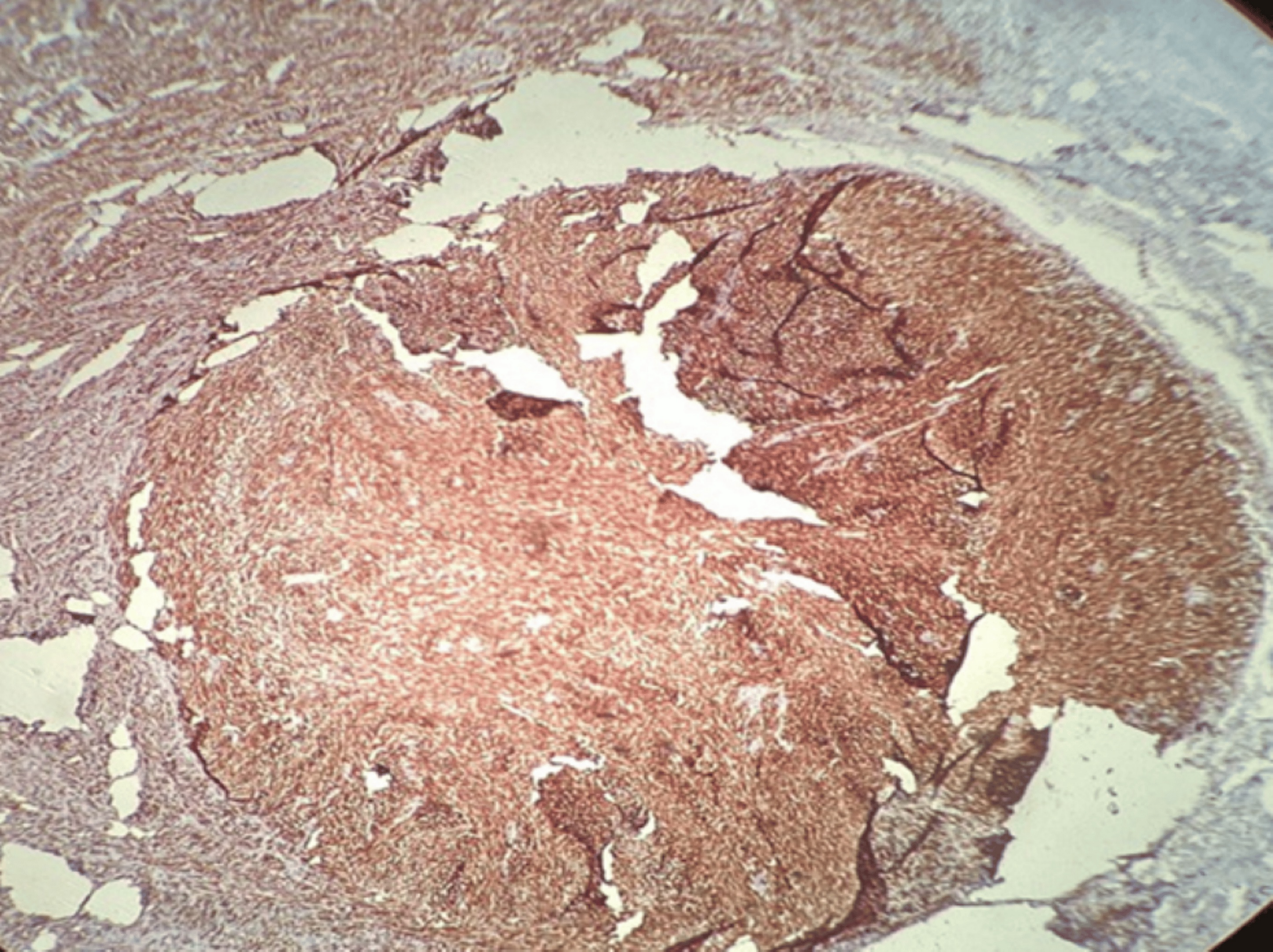
Immunostain for S-100 - positive in tumor cells showing brown cytoplasmic staining, 400x magnification

Immunohistochemistry (IHC) markers of these specimens were positive for human melanoma black (HMB-45) (Figure 5), negative for pancytokeratin (Figure 6), and positive for S-100 (Figure 7).

**Figure 8:**
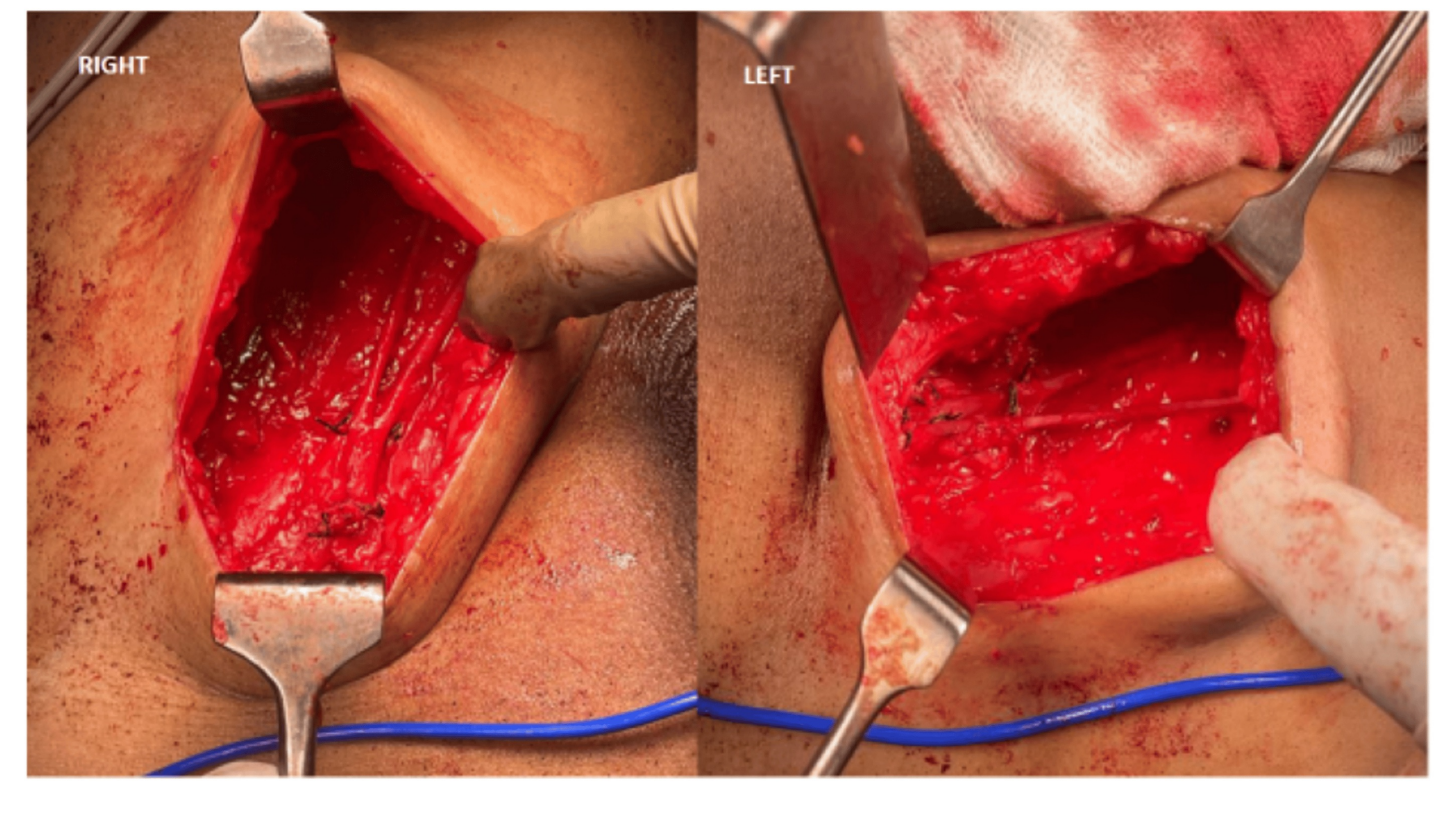
Right and left superficial inguinal LN dissection LN: lymph node

Since the proximal urethral margin was positive in partial penectomy specimens, and because of the aggressive nature of malignant melanoma histopathology, the patient was subjected to revision of the stump of the penis with bilateral superficial inguinal lymph node dissection (Figure 8).

The final histopathology report showed malignant melanoma with a proximal resected margin, and bilateral inguinal lymph node specimens were negative and tumor-free (stage I organ-confined).

After seeking an oncologist's opinion, the patient was started on a course of chemotherapy with cisplatin, bleomycin, vincristine, and thymosin immunotherapy. The patient does not show any recurrence or metastasis at the one-year follow-up.

## Discussion

PM, a rare cancer, accounts for under 0.2% of all male melanomas [2,8], predominantly occurring on the glans penis (82%), then the prepuce, urethral meatus, and shaft [8], peaking in the sixth to seventh decades [9,10]. Its advanced stages can cause dysuria, obstruction, hematuria, discharge, and sometimes melanic and fistula formation. Diagnosis is frequently delayed due to patient hesitation and diagnostic challenges [2,8]. It usually appears as a 1 cm pigmented papule, plaque, or ulceration, with differential diagnoses including junctional melanocytic nevus, penile melanosis, lentigo, and atypical pigmented macules [11]. These benign conditions are clinically similar to MM, making biopsy and histologic analysis essential for accurate diagnosis and avoiding excessive surgery. MM staging comprises stage I (limited to the penis), stage II (affects regional lymph nodes), and stage III (widespread) [12], with 43-60% of cases showing lymph node involvement at diagnosis [1-4]. The best treatment strategy is still debated, with some favoring aggressive surgical interventions [13].

Literature advocates for conservative penile surgery (local excision with a 3 to 5 cm margin or distal partial penectomy) when inguinal nodes are nonpalpable in patients with thin lesions (<1.5 mm) and recommend prophylactic superficial inguinal node dissection for lesions thicker than 1.5 mm^3^ [14,15]. Alternatively, sentinel lymphadenectomy using radiocolloid mapping and dye localization can avoid the morbidity of bilateral superficial inguinal node dissections while providing accurate staging for treatment and prognosis [15,16]. Chemotherapy is indicated for disseminated melanoma, with combination chemotherapy (Dacarbazine (DTIC), bis-chloroethyl nitrosourea (BCNU), cisplatin, and tamoxifen) yielding response rates of 15% to 45% [14-16].

Some literature noted that local excision or partial penile amputation with an adequate margin is effective for controlling T1 and T2 PMs. However, patients with proven metastases often succumb despite surgery and chemotherapy. Early-stage aggressive disease may be curable (Evidence Level 4) [17]. Literature also reported higher survival rates for patients undergoing wide local excision. Still, the prognosis remains poor due to delayed diagnoses and advanced disease characterized by suspicious inguinal crural adenopathy (Evidence Level 4) [18]. The patient prognosis for PM depends on the primary tumor stage and inguinal metastases presence [19]. Studies suggest that two-year and five-year survival rates are at 63% and 31%, respectively. All patients with nodal or distant metastases at diagnosis died within two years. The prognosis worsened significantly with ulceration, tumor depth ≥ 3.5 mm, or tumor diameter > 15 mm.

## Conclusions

PMs are rare but highly treatable with early surgical excision, given their resistance to chemotherapy and radiotherapy. Delays in diagnosis and surgery can worsen prognosis. Anamnesis, physical examination, and imaging studies are essential for improving patient survival. This case report underscores the effectiveness of conservative surgical treatment following recent guidelines, incorporating a 1 cm margin to prevent recurrence. Early detection is crucial for effective treatment in our patients. Early recognition, aggressive surgical intervention, and effective adjuvant therapy are vital for improving prognosis while preserving penile aesthetics and function.
